# Substrate Stiffness Modulates the Growth, Phenotype, and Chemoresistance of Ovarian Cancer Cells

**DOI:** 10.3389/fcell.2021.718834

**Published:** 2021-08-24

**Authors:** Yali Fan, Quanmei Sun, Xia Li, Jiantao Feng, Zhuo Ao, Xiang Li, Jiandong Wang

**Affiliations:** ^1^Department of Gynecologic Oncology, Beijing Obstetrics and Gynecology Hospital, Capital Medical University, Beijing, China; ^2^Chinese Academy of Sciences (CAS) Center for Excellence in Nanoscience, National Centre for Nanoscience and Technology, Beijing, China; ^3^Hospital of Beijing Forestry University, Beijing Forestry University, Beijing, China; ^4^Artemisinin Research Center, Institute of Chinese Materia Medica, China Academy of Chinese Medical Sciences, Beijing, China

**Keywords:** ovarian cancer, substrate stiffness, Yes-associated protein, epithelial-mesenchymal transition, chemoresistance

## Abstract

Mechanical factors in the tumor microenvironment play an important role in response to a variety of cellular activities in cancer cells. Here, we utilized polyacrylamide hydrogels with varying physical parameters simulating tumor and metastatic target tissues to investigate the effect of substrate stiffness on the growth, phenotype, and chemotherapeutic response of ovarian cancer cells (OCCs). We found that increasing the substrate stiffness promoted the proliferation of SKOV-3 cells, an OCC cell line. This proliferation coincided with the nuclear translocation of the oncogene Yes-associated protein. Additionally, we found that substrate softening promoted elements of epithelial-mesenchymal transition (EMT), including mesenchymal cell shape changes, increase in vimentin expression, and decrease in E-cadherin and β-catenin expression. Growing evidence demonstrates that apart from contributing to cancer initiation and progression, EMT can promote chemotherapy resistance in ovarian cancer cells. Furthermore, we evaluated tumor response to standard chemotherapeutic drugs (cisplatin and paclitaxel) and found antiproliferation effects to be directly proportional to the stiffness of the substrate. Nanomechanical studies based on atomic force microscopy (AFM) have revealed that chemosensitivity and chemoresistance are related to cellular mechanical properties. The results of cellular elastic modulus measurements determined by AFM demonstrated that Young’s modulus of SKOV-3 cells grown on soft substrates was less than that of cells grown on stiff substrates. Gene expression analysis of SKOV-3 cells showed that mRNA expression can be greatly affected by substrate stiffness. Finally, immunocytochemistry analyses revealed an increase in multidrug resistance proteins, namely, ATP binding cassette subfamily B member 1 and member 4 (ABCB1 and ABCB4), in the cells grown on the soft gel resulting in resistance to chemotherapeutic drugs. In conclusion, our study may help in identification of effective targets for cancer therapy and improve our understanding of the mechanisms of cancer progression and chemoresistance.

## Introduction

Epithelial ovarian cancer (EOC) is the leading cause of death among gynecological malignancies (Walker et al., 2015). This poor prognosis is mainly because most patients are diagnosed at a late stage and have drug resistance. The standard treatment of EOC is the surgical removal of the tumor tissue followed by chemotherapy. Cisplatin and paclitaxel represent the two most widely used first-line agents for EOC. Most EOC patients are chemotherapy-sensitive; however, 15% experience primary platinum resistance (Jemal et al., 2010). Many patients also experience cancer recurrence within 2 years of initial treatment due to acquired resistance to platinum-based chemotherapy. Five-year survival is only 30% in advanced EOC (Mor and Alvero, 2013). Chemotherapy resistance is considered to be a major obstacle to the successful treatment of EOC. This highlights the need to elucidate mechanisms that drive cancer progression and resistance and to develop therapeutic strategies for drug-resistant relapses.

The role of tissue stiffness has been explored in various human cancers. Many factors promote tumor tissue stiffening, including extracellular matrix (ECM) remodeling and an increase in the interstitial pressure due to tumor growth and chaotic microvasculature (Erkan et al., 2012). ECM is primarily composed of collagen and fibrous proteins, proteoglycans. ECM stiffening in tumors is caused by the reorganization of the stroma by the excessive activation of ECM enzymes and proteins that covalently cross-link collagen fibers and other ECM components (Levental et al., 2009). Cells sense and respond to the mechanical properties of the ECM through mechanocellular systems, including focal adhesion complexes, integrins, the actin cytoskeleton, and associated molecular motors. Recent reports indicate that mechanical forces are also directly transmitted from the ECM to the nucleus by the physical anchoring of the cytoskeleton to the nuclear lamina (Tajik et al., 2016). The Hippo pathway effector protein Yes-associated protein (YAP) is a well-known intracellular transducer of mechanical stimuli (Dupont et al., 2011). Studies have shown that the Hippo pathway and YAP not only respond to mechanical cues but are also important mediators of cellular responses to these stimuli (Dupont et al., 2011; Aragona et al., 2013). In addition to providing structural support, the ECM can regulate cellular behavior. Recently, studies have reported that the stiffness of the ECM plays a pivotal role in tumor initiation, progression, metastasis, and therapeutic efficacy (Sethi et al., 1999; Feng et al., 2013; Liu et al., 2016).

Cancer cells gain chemoresistance through a variety of mechanisms. Apart from intrinsic resistance factors (Galluzzi et al., 2012), tumor chemoresistance is also affected by the biochemical and physical properties of the tumor microenvironment (TME) (Ostman, 2012; Kharaishvili et al., 2014; Wu et al., 2021). The physical components of the TME, such as high interstitial fluid pressure and densely packed cells, hinder drug delivery (Correia and Bissell, 2012). Cancer cells can acquire chemoresistance via cell-cell and cell-ECM interactions (Landowski et al., 2003). When a cancer cell comes into close contact with the stromal cells or ECM, adhesion induces the production of pro- and anti-apoptotic molecules (Hazlehurst et al., 2000, 2007). Mounting evidence suggests that not only the composition of the ECM but also its stiffness can significantly affect chemoresistance (Schrader et al., 2011; Liu et al., 2015). Drug resistance arises from multiple mechanisms, such as drug target mutations, drug metabolism, drug efflux, etc. Recently, epithelial-mesenchymal transition (EMT) has received increasing attention for its role in cancer drug resistance. Several studies show that cancer cells resistant to cisplatin and/or paclitaxel had acquired a mesenchymal phenotype (Kajiyama et al., 2007; Yang et al., 2014), which points to EMT as a possible driver of resistance.

Instead of the classic patterns of metastasis via the hematogenous route and extravasation at a distal site, ovarian cancer cells (OCCs) often metastasize through the intraperitoneal fluid to the omentum, retroperitoneal lymph nodes, and even to the parenchyma of the liver or lungs. The prognosis of patients with EOC is most likely related to the degree of peritoneal dissemination. The omentum, which is one of the most frequent sites of ovarian cancer metastasis, is predominantly composed of adipose tissue. Adipocytes are the key components of ovarian cancer TME and have been shown to stimulate the migration of OCCs toward omentum through secreted cytokines such as IL-8 (Nieman et al., 2011). Omental adipocytes also have been shown to produce fatty acids, which can be used as an energy source, to support OCCs proliferation. Adipocytes also influence OCC chemoresistance. Activated Akt enhances the survival of OCCs and promotes the chemoresistance through attenuating p53 proapoptotic signaling (Yang et al., 2006; Fraser et al., 2008). Studies have demonstrated that adipocyte-secreted arachidonic acid (AA) acts directly on OCCs to activate AKT and inhibit cisplatin-induced apoptosis (Yang et al., 2019). Currently, there is a gap in our understanding of the effects of substrate stiffness on OCC chemoresistance and the driving mechanisms that underlie it. In the present study, we utilized a collagen-coated polyacrylamide hydrogel system with elastic properties that mimic those of tumor, and metastatic target tissues for OCC dissemination, to investigate the role of substrate stiffness in OCC growth and chemotherapeutic response.

## Materials and Methods

### Cell Culture

The EOC cell line, SKOV-3 (American Type Culture Collection [ATCC]) was cultured in RPMI-1640 media (Gibco, Waltham, MA, United States) supplemented with 10% fetal bovine serum (Corning, New York, NY, United States), 100 U mL^–1^ penicillin, and 100 mg mL^–1^ streptomycin (all from Hyclone, Logan, UT, United States) in a humidified incubator at 37°C with 5% CO_2_.

### Proliferation and Cytotoxicity Analysis

For proliferation analysis, the OCCs were seeded in 24-well culture plates, coated with hydrogel substrates of different stiffness (Matrigen, United States), at a concentration of 2,500 cells/cm^2^, and cultured for 6 days. Cell viability was determined once every 24 h. For cytotoxicity analysis, SKOV-3 cells were seeded in 96-well culture plates, coated with hydrogel substrates of different stiffness (Matrigen, United States), at a concentration of 4 × 10^3^ cells/well for 24 h. The cells were then treated with varying concentrations of paclitaxel (Sigma, United States) or cisplatin (Tokyo Chemical Industry, Japan) for 48 h. The concentrations of paclitaxel were 0, 10^–4^, 10^–3^, 10^–2^, 10^–1^, 1 μg/mL. The concentration of cisplatin were 0, 10^–2^, 10^–1^, 1, 5, and 10 μM. After discarding the supernatant, Cell Counting Kit-8^TM^ (CCK-8) (DOJINDO, Japan) working solution was added to each well and incubated for 3 h at 37°C. The absorbances at 450 nm were read using a spectrophotometer (NanoDrop, ND-100, United States). Each experiment was repeated three times to assess for the consistency of the results.

### Environmental Scanning Electron Microscopy

The SKOV-3 cells were cultured on small circular glass sheets coated with hydrogel substrates of different stiffness (Matrigen, United States). The cells were cultured in this environment for three days. Next, the cells were washed with phosphate-buffered saline (PBS) three times and fixed using 2.5% glutaraldehyde for 30 min at 25°C. The cells were then again washed with PBS three times. Next, the cells were dehydrated using 30, 50, 70, 85, 95, and 100% ethanol for 15 min each time. A carbon dioxide critical point dryer was used to replace the ethanol in the sample for drying. Finally, the cells were imaged using the low vacuum mode of the environmental scanning electron microscope (Quanta 200 FEG, FEI, United States).

### Immunofluorescence Staining

The SKOV-3 cells were seeded on small circular glass sheets, coated with hydrogel substrates of different stiffness (Matrigen, United States) and were cultured for 48 h. The cells were washed with PBS three times and then were fixed using 4% paraformaldehyde solution for 30 min. After washing with PBS three times, the cells were treated with the 2.5% Triton-X^TM^ for 30 min. The cells were blocked with 3% bovine serum albumin (BSA) (Solarbio, China) and were incubated with a 1:100 dilution of primary antibody against α-tubulin (DM1A-3873s, Cell Signaling Technology, United States), paxillin (sc-365379, Santa Cruz, United States), vimentin (sc-6260, Santa Cruz), E-cadherin (sc-21791, Santa Cruz), β-catenin (sc-7963, Santa Cruz), YAP (sc-101199, Santa Cruz) overnight at 4°C. The cells were then washed with PBS three times and incubated with a secondary antibody (ab150113, Abcam, United Kingdom) for 1 h. 4′,6-diamidino-2-phenylindole (DAPI) (Cell Signaling Technology), which was diluted with PBS to 1 μg/mL, was added and incubate for 5 min, then washed with PBS three times. Finally, images were acquired using a laser scanning confocal microscope (UltraVIEW VoX; PerkinElmer, United States).

### Western Immunoblotting

The SKOV-3 cells were seeded in 6-well culture plates, coated with hydrogel substrates of different stiffness (Matrigen, United States), at a concentration of 2.5 × 10^5^ cells per well and were cultured for 72 h. Cell lysates were prepared in radioimmunoprecipitation assay (RIPA) buffer plus PhosStop^TM^ (Biorigin, BN25015). Equal amounts of protein were separated by gel electrophoresis and transferred onto a polyvinylidene fluoride (PVDF) (Merck Millipore Ltd., Tullagreen, Carrigtwohill) membrane. The membrane was blocked with 5% non-fat dry milk and then incubated with a 1:1,500 dilution of primary antibody against α-tubulin (DM1A-3873s, Cell Signaling Technology), paxillin (D9G12-12065, Cell Signaling Technology), vimentin (D21H3-5741, Cell Signaling Technology), E-cadherin (24E10-3195, Cell Signaling Technology), β-catenin (D10A8-8480, Cell Signaling Technology), YAP (sc-101199, Santa Cruz), ABCB1 (ABP59326, Abbkine), or ABCB4 (ABP59247, Abbkine) overnight at 4°C. The membrane was then washed and incubated with a secondary peroxidase-conjugated antibody (ab-150077 or ab150113, Abcam) for 1 h after washing. Antibody binding was detected using an enhanced chemiluminescence detection buffer from Alpha Innotech Imaging System (San Leandro, CA, United States). ImageJ software (Fiji-win64) was used to analyze the data. Each experiment was repeated three times to assess for consistency of results.

### Atomic Force Microscopy

The SKOV-3 cells were seeded in Petri dishes, coated with hydrogel substrates of different stiffness (Matrigen, United States) for 3 days. To probe the nanomechanical properties of the cells, we employed an AFM instrument (5,500; Keysight, Santa Rosa, CA, United States) combined with an inverted microscope (TE2000U; Nikon, Tokyo, Japan). As the 0.5 kPa hydrogel was too soft for AFM experiments, we instead used hydrogels with stiffness values of 4, 25, and 50 kPa for this test. Each cell was probed by recording the approach part of the force-distance curve at the central region of the cytoplasm with a frequency of 1 Hz. According to our previous method (Shi et al., 2010), silica microspheres (Thermo Fisher Scientific, United States) with a diameter of approximately 10 μm were attached to the tipless cantilever with a typical spring constant (k) of about 0.2 Nm^–1^ (TL-CONT, NANOSENSORS, Neuchatel, Switzerland). The force-distance curves were converted into force-indentation curves and fitted to the spherical Hertz model to calculate Young’s modulus of the cells. Before AFM indention testing, the spring constant of the cantilever was determined using the thermal tune method. According to the slope of the force-distance curves acquired on the glass substrate, the deflection sensitivity of the cantilever was measured in the fluid.

### mRNA Expression Analysis

High throughput sequencing was used to identify significant differences in gene expression in the cells grown on different substrates. The total RNA from each sample was isolated using TRIzol^®^ reagent (Merk, Darmstadt, Germany). The triplicate samples of all assays were constructed an independent library. TruSeq RNA Sample Prep Kit (Illumina, FC-122-1001) was used with 1 μg of total RNA for the construction of sequencing libraries. NEBNext^®^ Poly(A) mRNA Magnetic Isolation Module kit was used to enrich the poly (A) tailed mRNA molecules from 1 μg total RNA. The gene expression analysis were performed to analyze the differentia expression genes (DEGs) between samples. Next, pathway analysis was applied to determine the significant pathways of the differential genes using the Kyoto Encyclopaedia of Genes and Genomes (KEGG) database. The *P*-values for the pathways of all the differential genes were calculated. *P*-value were used to carry out significance analysis. Parameters for classifying significantly DEGs are ≥ 2-fold differences (|log2FC| ≥ 1, FC: the fold change of expressions) in the transcript abundance and *P* < 0.05.

### Statistics Analysis

Origin Pro (version 8.5, OriginLab Corporation, Northampton, MA, United States) was used to perform statistical analysis with a one-way analysis of variance (ANOVA). A two-sample *t*-*t*est was used to determine statistical significance. Differences with a *p*-value below 0.05 were considered statistically significant for all experiments unless otherwise specified.

## Results

### Substrate Stiffness Influences the OCC Proliferation

**FIGURE 1:**
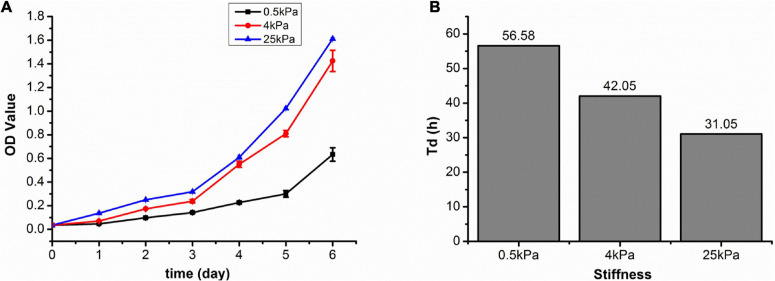
**(A)** SKOV-3 cell growth profiles on substrates of varying stiffness; **(B)** doubling time (Td) of SKOV-3 cells on different substrates after 72 h.

The most common sites of metastasis for OCCs are the peritoneum, lymph nodes, lungs, and liver. According to previous reports, hydrogels with stiffness of 0.5, 4, and 25 kPa are equivalent to the stiffness of lymph nodes, peritoneum, and tumor tissues (Levental et al., 2007; McKenzie et al., 2018). Growth profiles of SKOV-3 cells cultured on different substrates were plotted by calculating cell numbers in 24 h intervals for 6 days. The SKOV-3 cells in the 0.5, 4, and 25 kPa hydrogels entered the logarithmic growth phase at the 5th, 4th, and 4th day, respectively (Figure 1A). The doubling time (Td) results based on 72 h cultures showed that the SKOV-3 cells in the 0.5, 4, and 25 kPa hydrogel substrates had a Td of 56.58, 42.05, and 31.05 h, respectively (Figure 1B). These results indicated that the proliferation rate of SKOV-3 cells was increased when on a more rigid substrate.

### Substrate Stiffness Influences the Skeleton of Ovarian Cancer Cell SKOV-3

**FIGURE 2:**
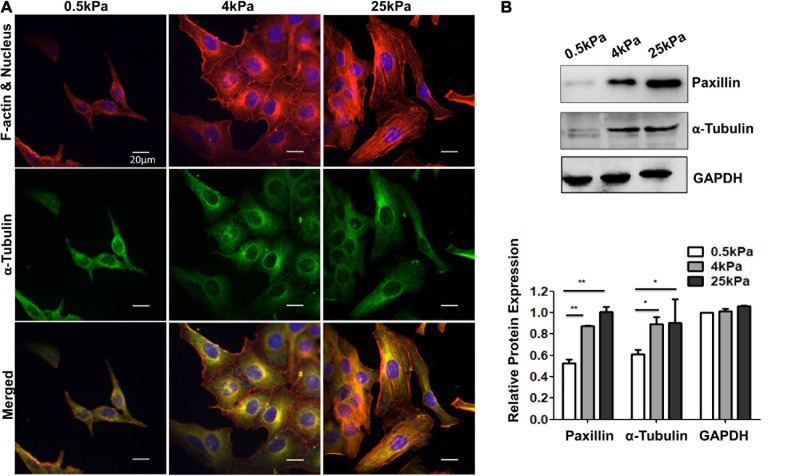
**(A)** Structures of F-actin and microtubulin in SKOV-3 cells grown on substrates of different stiffness. **(B)** Expression patterns of microtubulin and paxillin in MCF-7 cultured on different substrates for 48 h. *P < 0.05 and **P < 0.01.

Cells adhere to the ECM through focal adhesions that link the actin cytoskeleton to ECM. The cytoskeleton is a dynamic network composed mainly of three kinds of fiber structures, namely F-actin, microtubules, and intermediate filaments. The actin cytoskeleton is known to be highly responsive to mechanical stresses (Fletcher and Mullins, 2010). The cytoskeleton structures of SKOV-3 cells on the tested substrates were investigated using laser scanning confocal microscopy. The results showed that the fluorescence of F-actin and tubulin were weak in cells on soft substrates, whereas the cells on rigid substrates exhibited prominent stress fibers (Figure 2A). Additionally, the western blot assays showed that the expression of tubulin and focal adhesion-paxillin increased as substrate stiffness also increased (Figure 2B). These results indicated that the cytoskeleton was remodeled and reinforced to match the force applied by the different substrates.

### Substrate Stiffness Affects Cell Stiffness

**FIGURE 3:**
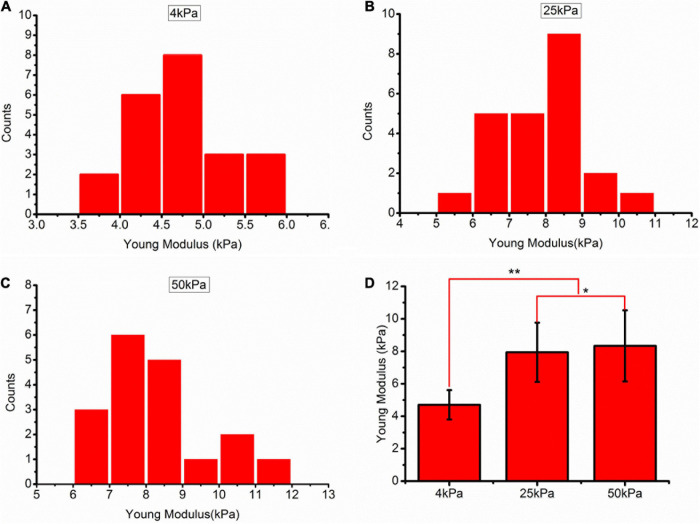
**(A–C)** Frequency distribution of the Young’s modulus of SKOV-3 cells cultured on hydrogels with different stiffness for 3 days. **(D)** Medians of the Young’s modulus. *P < 0.05 and **P < 0.01.

Mechanical analysis of the force-distance curves was performed to determine the relative cell elasticities indicated by Young’s modulus. The curves were then fitted to the spherical Hertz model. AFM measurements demonstrated that Young’s modulus of SKOV-3 cells grown on soft substrates was less than that of cells grown on stiff substrates (*p* < 0.05; Figure 3). Values are expressed at mean ± standard deviation. The values of Young’s modulus in the 4, 25, and 50 kPa groups were 4.70 ± 0.91, 7.94 ± 1.82, and 8.34 ± 2.19 kPa, respectively.

### Substrate Stiffness Regulates the Phenotypes of OCCs

**FIGURE 4:**
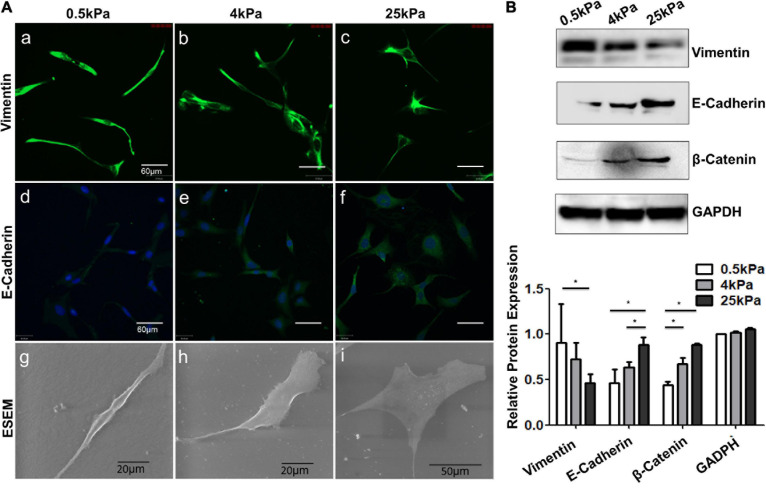
Role of substrate stiffness in epithelial-mesenchymal transition (EMT) induction. **(A)** Laser confocal microscopy and environmental scanning electron microscopy (ESEM) of SKOV-3 cells on substrates of varying stiffness. **(B)** Western blotting showing expression levels of vimentin, E-cadherin, and β-catenin. *P < 0.05.

Characteristic phenotypic markers of mesenchymal and epithelial cells were assessed to demonstrate EMT across the cell population. These included an increase in vimentin expression, decreases in E-cadherin and β-catenin expression, and a more elongated cell shape. Immunofluorescence staining and Western blotting showed that SKOV-3 cells grown on stiffer substrates had increased vimentin expression and decreased E-cadherin and β-catenin expression (Figures 4Aa–f,B). Cell shape was also observed to change with stiffness. Images were acquired using an environmental scanning electron microscopy in low vacuum mode to observe the morphological response to changes in substrate stiffness (Figures 4Ag–i). The shapes of cells on the soft substrates (0.5 kPa) were mostly spindle-like and the cells were well spread out and flattened on stiffer substrates. The shape of cells grown on the softer substrates showed characteristics of epithelial cells. These results indicated that EMT occurred in OCCs on the soft substrates.

### Substrate Stiffness Promotes YAP Nuclear Localization

**FIGURE 5:**
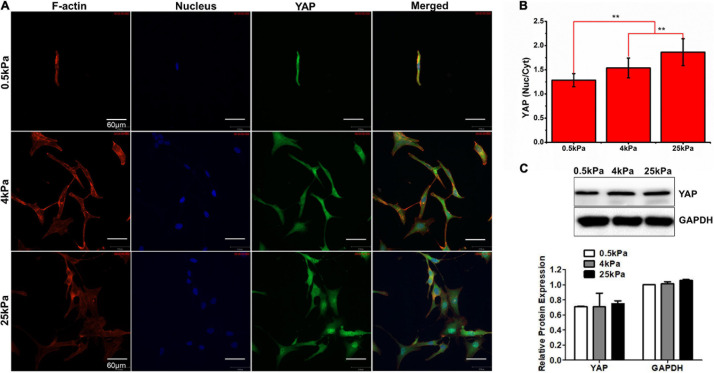
**(A)** Characterization of YAP fluorescence and stress fibers in SKOV-3 cells grown on substrates with different stiffness. **(B)** Quantification of the nuclear/cytoplasmic ratio of YAP. **(C)** Western blotting showing the expression of YAP. **P < 0.01.

YAP is the main effector of the Hippo signaling pathway and is involved in signal transduction and transcriptional activation of downstream target factors (Piccolo et al., 2014). YAP was noted as an oncogene in previous studies. High YAP activity drives proliferation, differentiation, invasion, and metastases of cancer cells. Studies have revealed that, apart from the Hippo pathway, YAP is regulated by mechanical forces (Benham-Pyle et al., 2015). Therefore, we investigated the effect of substrate stiffness on YAP nuclear localization in OCCs. The immunofluorescence studies showed that the nuclear distribution of YAP in SKOV-3 cells grown on stiff substrates was elevated compared to cells grown on soft substrates (*p* < 0.01; Figures 5A,B). Western blotting showed that there were no significant differences in the expression of YAP among the cells in the three substrate rigidity groups (Figure 5C). These results demonstrated that substrate stiffness promoted nuclear translocation of YAP. Substrate stiffness regulates the chemosensitivity of OCCs.

**FIGURE 6:**
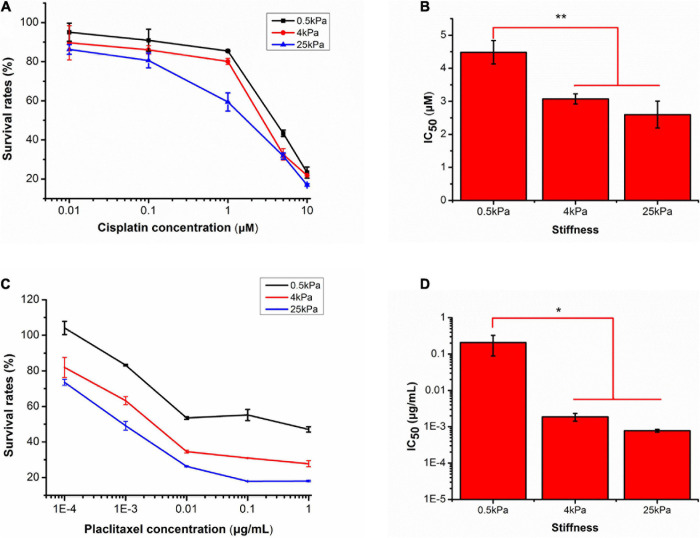
Responses of SKOV-3 cells grown on different substrates to cisplatin and paclitaxel. **(A)** Survival rates of SKOV-3 cells on different substrates after treatment with different concentrations of cisplatin. **(B)** IC50 values of cisplatin on different substrates. **(C)** Survival rates of SKOV-3 cells on different substrates after treatment with different concentrations of paclitaxel. **(D)** IC50 values of paclitaxel on different substrates. *P < 0.05 and **P < 0.01.

The chemotherapeutic drugs cisplatin and paclitaxel work based on distinct molecular mechanisms. Cisplatin forms DNA cross-links and platinum adducts between DNA and proteins, which causes DNA damage and subsequent cell death (Florea and Büsselberg, 2011). Paclitaxel induces cell death by binding to microtubulin, thus causing microtubule dysfunction, induction of cell cycle arrest in G2-M, and activation of apoptosis (Horwitz, 1992). The pharmacological responses of the SKOV-3 cells on different substrates to the antitumor drugs cisplatin and paclitaxel were evaluated. Values are expressed at mean ± standard deviation. For cisplatin at the lowest concentration (less than 1 μM), there were no significant differences in survival rates of SKOV-3 cells on different substrates. At 1 μM of cisplatin, survival rates of SKOV-3 cells on the 0.5, 4, and 25 kPa substrates were 79.18 ± 10.84, 81.77 ± 3.06, and 57.99 ± 4.10%, respectively. There was no significant difference between 0.5 and 4 kPa, but there was a significant difference between the former two and the 25 kPa substrate (*p* < 0.05). However, at 5 μM, the cell survival rates were 46.48 ± 5.54, 31.08 ± 3.30, and 33.63 ± 3.43%, respectively. There was no significant difference between 4 and 25 kPa, but there was a significant difference between the latter two and 0.5 kPa (*p* < 0.05). At 10 μM, there were no significant differences in survival rates of SKOV-3 cells on different substrates (Figure 6A). Subsequently, the mean IC_50_ values for cisplatin were 4.48 ± 0.35, 3.07 ± 0.15, and 2.60 ± 0.41 μM for the 0.5, 4, and 25 kPa substrates, respectively (Figure 6B). There was a significant difference between 0.5 kPa and the latter two (*p* < 0.01), but there was no significant difference between 4 and 25 kPa substrate (*p* < 0.05).

For paclitaxel, the effects of substrate stiffness on pharmacological response were similar to that of cisplatin. As the concentration of paclitaxel increased, the cell survival rates on all substrates gradually decreased. At a concentration of 10^–4^ μg mL^–1^, the cell survival rates on the 0.5, 4, and 25 kPa hydrogels were 101.47 ± 11.98, 78.81 ± 6.69, and 71.55 ± 3.72%, respectively. There was a significant difference between 0.5 kPa and the latter two (*p* < 0.05), but there was no significant difference between 4 kPa and 25 kPa substrate (*p* < 0.05). When increased to 10^–3^ μg mL^–1^, the cell survival rates were 81.99 ± 2.15, 61.25 ± 3.87, and 50.89 ± 3.51%, respectively. There was a significant difference between 0.5 and 4 kPa (*p* < 0.01), between 4 and 25 kPa (*p* < 0.05). At 10^–2^ μg mL^–1^, the cell survival rates were 52.18 ± 2.31, 34.2 ± 0.84, and 28.24 ± 3.40%, respectively. There was a significant difference between 0.5 and 4 kPa (*p* < 0.01), and there was no significant difference between 4 and 25 kPa. At the highest dose of 10^–1^ μg mL^–1^, the cell survival rates of the cells grown on the 0.5, 4, and 25 kPa substrates was 51.27 ± 7.11, 29.97 ± 1.72, and 18.06 ± 0.3%, respectively. There was a significant difference between 0.5 and 4 kPa (*p* < 0.01), between 4 and 25 kPa (*p* < 0.05). At the highest dose of 1 μg mL^–1^, the cell survival rates of the cells grown on the 0.5, 4, and 25 kPa substrates was 51.48 ± 7.14, 29.02 ± 2.49, and 16.97 ± 1.85%, respectively (Figure 6C). There was a significant difference between 0.5 and 4 kPa (*p* < 0.01), but there was no significant difference between 4 and 25 kPa substrate (*p* < 0.05). The mean IC_50_ values of paclitaxel for cells grown on the 0.5, 4, and 25 kPa substrates were 0.21 ± 0.12, 0.0018 ± 0.0004, and 0.0007 ± 0.00005 μg mL^–1^, respectively (Figure 6D). There was a significant difference between 0.5 kPa and the latter two, but there was no significant difference between 4 kPa and 25 kPa substrate (*p* < 0.05).

### Analysis of SKOV-3 Cell Gene Expression When Grown on Different Substrates

**FIGURE 7:**
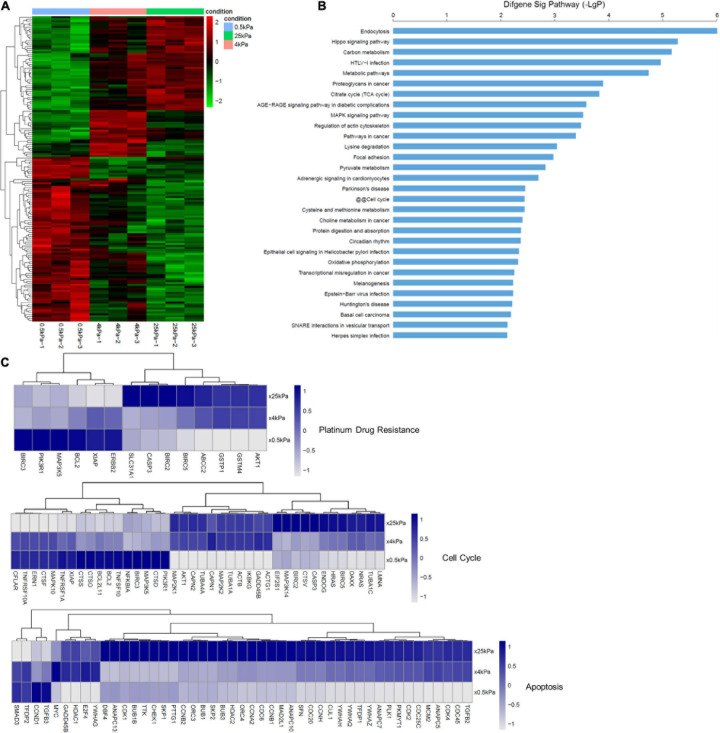
Substrate stiffness alters the expression of genes. **(A)** Changes in the SKOV-3 cell gene expression when cultured on substrates with different stiffness. **(B)** Significant pathways of differentially expressed genes in SKOV-3 cells on different substrates. **(C)** Expression levels of selected genes whose human orthologs are involved in platinum-based drug resistance, cell cycle, and apoptosis.

In order to identify the effect of substrate stiffness on gene expression, we evaluated for differentially expressed mRNAs in the SKOV-3 cells grown on the different substrates via microarray analysis. Changes in gene expression were measured after 3 days of growth on the gels. The hierarchical clustering analysis of these changes in mRNA expression is shown in Figure 7A. We found that the general direction of changes in gene expression (up or downregulated) was more similar between cells cultured on 25 and 4 kPa substrates. We performed the pathways analyses of the differentially expressed mRNA according to the KEGG database. Figure 7B lists the 30 most significant signaling pathways. Among these, endocytosis, the Hippo signaling pathway, metabolic pathways, proteoglycans in cancer, the MAPK signaling pathway, regulation of actin cytoskeleton, focal adhesion, and cell cycle were ranked the highest. Figure 7C expression levels of genes involved in platinum drug resistance, apoptosis and cell cycle. Many genes whose molecular action imparts in apoptosis were downregulated in the cells grown on 0.5 kPa substrate. Platinum drug-resistance genes including ERBB2, BCL2, MAP3K5, PIK3R1, and BIRC3 were significantly upregulated in SKOV-3 cells on soft substrates.

### Substrate Stiffness Affects the Expression of Multidrug Resistance Proteins

**FIGURE 8:**
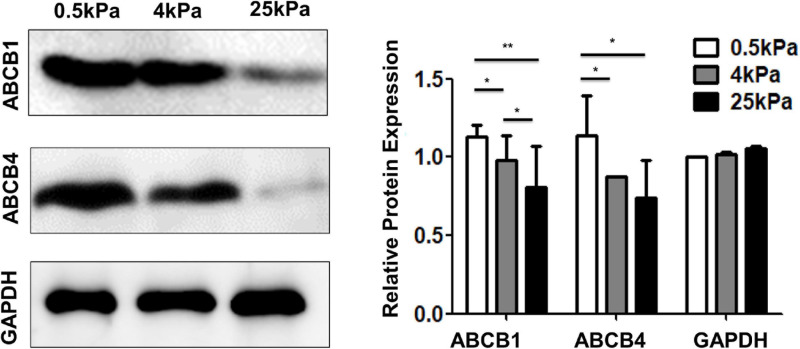
Substrate stiffness affects the expression of multidrug resistance proteins.

The ABC transporter (ATP-binding cassette transporter) superfamily is a group of transmembrane proteins and transporters that cause drug resistance by using ATP to excrete multiple anticancer drugs. After tumor cells develop resistance to a drug, they also develop resistance to drugs with different structures and mechanisms of action that they have not been exposed to, which is a phenomenon called multidrug resistance (MDR). Overexpression of ABC transporters is an important cause of MDR. Among the various ABC transporters, ABCB1 (MDR1) and ABCB4 (MDR3) are thought to play important roles in ovarian cancer. The results showed that the expression of ABCB1 and ABCB4 on the soft substrates was higher than that on rigid substrates (Figure 8). These results indicated that substrate stiffness affected the expression of genes related to multidrug resistance.

## Discussion

In this study, we demonstrated that the growth and the chemotherapeutic response of OCCs *in vitro* are markedly affected by substrate stiffness. It has previously been demonstrated that substrate stiffness can regulate proliferation in a variety of other cancer cells (Tilghman et al., 2010). In our study, the growth profiles of the human OCC line SKOV-3 were analyzed and the results showed increased proliferation and rapid cell cycle progression when cells were cultured on rigid substrates. In previous studies, it has been demonstrated that the expression of cyclin-D was upregulated and critical mitogenic signaling was promoted on rigid substrates (Schrader et al., 2011; Hui et al., 2017). YAP was noted as an oncogene in previous studies. The canonical mechanism of YAP regulation is a phosphorylation cascade in the Hippo pathway (Huang et al., 2005). YAP is inhibited through direct phosphorylation of the central components of the Hippo pathway including the Mst1/2 and Lats1/2 kinase cascades (Piccolo et al., 2014). Phosphorylated YAP localizes to the cytoplasm in an inactive state and dephosphorylated YAP enters the nucleus from the cytoplasm. In the nucleus, YAP mainly induces cell proliferation and the expression of anti-apoptotic genes by interacting with various transcription factors such as the TEA region (TEAD) family. Besides the canonical Hippo pathway, investigators have recently uncovered that YAP is regulated by different types of mechanical stimuli including physical stretching (Benham-Pyle et al., 2015), ECM topography (Aragona et al., 2013), and stiffness (Dupont et al., 2011) via Hippo-independent mechanisms. Here, we used confocal microscopy to visualize and quantify YAP localization in SKOV-3 cells grown on hydrogels of differing stiffness. The results showed that the levels of nuclear localization were positively correlated with the stiffness of the hydrogel substrates. Our data agree with similar findings from other studies linking YAP nuclear localization to greater proliferation on stiffer substrates. In the literature, cells cultured on rigid substrates showed high nuclear localization and elevated transcriptional activities of YAP, whereas, in cells that were gown on softer substrates, YAP translocated to the cytoplasm and was inactivated (Zhao et al., 2012; Totaro et al., 2017). Studies suggest that the inhibition of YAP interaction with its transcriptional partners is a potential strategy for cancer therapy. Verteporfin (VP), used clinically for the treatment of macular degeneration, has been proven to inhibit the interaction between YAP and TEAD and has shown potential as an anticancer treatment. Liu-Chittenden et al. (2012) showed that VP could disrupt the TEAD-YAP association and inhibit YAP-induced liver overgrowth. It was also observed that VP could effectively reduce proliferation and inhibit the growth of OCCs *in vivo* and *in vitro* (Feng et al., 2016). Our findings combined with those of the previously mentioned studies indicate that targeting YAP signaling may be a promising therapy strategy against stiffness-induced proliferation.

AFM-based nanomechanical studies have revealed that cellular mechanical properties are closely related to certain disease states. Many studies have reported that cancer cells are physically softer than normal cells (Lekka, 2016; Alibert et al., 2017) and metastatic cancer cells are more mechanically compliant than their non-metastatic counterparts (Li et al., 2008; Xu et al., 2012; Park, 2016). Recent AFM experiments investigating the mechanical properties of chemo-sensitive and resistant cancer cells have contributed to unraveling the multifaceted nature of chemotherapeutic resistance. Sharma et al. (2014) demonstrated that cisplatin-resistant OCCs (OVCAR5-CisR and SKOV3-CisR) were much stiffer than their cisplatin-sensitive counterparts (OVCAR5 and SKOV-3, respectively). Moreover, their results indicated that cisplatin resistance correlates with the dynamic reorganization of actin filaments and nano-mechanical compliance (Sharma et al., 2014). Studies have shown that paclitaxel and cisplatin induce an increase in the stiffness of cancer cells (Ren et al., 2015). In addition, cisplatin-sensitive OCCs (A2780) showed a dose-dependent increase in cell stiffness after cisplatin treatment, while resistant cells (A7890cis) were unaffected. However, one study showed that cisplatin- and paclitaxel-resistant OCCs were softer than drug-sensitive cells (Kapoor et al., 2018). Seo et al. (2015) found that there was a bimodal distribution in the histogram of mechanical stiffness. This bimodal distribution implies the existence of two different subpopulations. The peak of the lower stiffness almost overlapped with the average mechanical stiffness of sensitive cells. All these findings demonstrated that cellular nano-mechanical studies help to reveal how cancer cells acquire drug resistance. However, most of these experiments were performed on plastic Petri dishes, whose mechanical properties are very different from those of natural tissues. The elastic properties of cells are mainly affected by their intracellular actin cytoskeleton. Cytoskeletal reorganization is responsible for changes in cellular elastic modulus. In addition, microtubules and intermediate filaments such as vimentin and keratin play crucial roles in the elastic properties of cells (Wu et al., 1998; Beil et al., 2003). Our study demonstrated that the organization and expression of various cytoskeleton components, including F-actin, microtubulin, and vimentin, varied with changes in substrate stiffness. Our results showed that OCCs grown on softer substrates with a lower elastic modulus and were less sensitive to chemotherapeutic agents. The literature regarding the response to mechanical changes of cancer cells and their normal counterparts to different gel substrate stiffnesses reported that cancer cells were less susceptible to changes in substrate when substrate stiffness was increased compared to their normal counterparts. Our study, combined with those previously mentioned, indicates that research into the effect of different substrate stiffness on the mechanical properties of drug-sensitive and drug-resistant cancer cells is of great significance in understanding the underlying mechanisms of chemoresistance from a nanomechanical perspective.

The EMT is a process by which epithelial cancer cells lose cell-cell adhesion, apical-basal polarity, and acquire a spindle-like morphology. Epithelial markers such as E-cadherin, cytokeratins, and occludin are downregulated. Meanwhile, mesenchymal markers such as N-cadherin and vimentin are upregulated (Thompson et al., 2005; Taube et al., 2010). EMT can be activated not only by a variety of signaling pathways such as TGF-β, EGF, miRNA, AKT, and PI3K but also by changes in ECM stiffness and endogenous mechanical stress (De Craene and Berx, 2013). It has been previously demonstrated that substrate stiffness can promote EMT in multiple cancer cell lines (Schrader et al., 2011; Rice et al., 2017). The stiffness of metastatic microenvironments is significantly lower than that of the primary tumor tissue. In this study, we utilized polyacrylamide hydrogels with elastic properties that mimic tumor tissue and tissues commonly metastasized by OCCs to investigate the role of ECM stiffness in promoting OCC malignancy. We have demonstrated that changes in cellular morphology and the expression of a variety of molecules were all indicative of EMT following culture on gels of lower rigidity. The cellular morphology changed to a spindle-like shape, while epithelial markers including E-cadherin and β-catenin were downregulated, and mesenchymal markers such as vimentin were upregulated in softer environments. Our findings suggest that a reduction in the stiffness of the cancer cell niche, as would be encountered by disseminated or metastatic OCCs, would be sufficient to promote EMT.

In the context of cancer, the EMT mechanism is crucial for cancer initiation and metastasis. Growing evidence demonstrates that apart from contributing to cancer progression, EMT can promote chemotherapy resistance in OCCs. The epithelial marker E-cadherin is downregulated and the mesenchymal marker vimentin is upregulated in paclitaxel-resistant epithelial OCCs (NOS-PR, TAOV-PR, and SKOV-3) (Kajiyama et al., 2007). It was demonstrated that an increase in the expression of miR-181a-induced EMT in OCCs mediated resistance to paclitaxel-based therapies (Li et al., 2016). Twist1 is a highly evolutionally conserved basic Helix-Loop-Helix transcriptional factor (bHLH). Many studies have highlighted the role of in promoting cancer cell EMT (Watanabe et al., 2004; Yang et al., 2004). A study showed that miR-186 regulation of Twist1 can be seen as a promising strategy to sensitize OCCs that have undergone EMT and chemotherapy-induced resistance (Zhu et al., 2016). Recent studies have demonstrated that cancer cells acquire chemoresistance based on the substrate stiffness-induced EMT. Rice et al. (2017) demonstrated that substrate stiffness induced EMT and promoted chemoresistance in pancreatic cancer cells. Our results suggest that a stiff environment promotes epithelial phenotypes in OCCs, whereas low stiffness induces mesenchymal phenotypes. We further investigated whether EMT affected OCC susceptibility to chemotherapy. We found that the chemosensitivity of SKOV-3 cells to cisplatin and paclitaxel decreased as the substrate softened. Our findings highlighted that substrate stiffness plays an important role in EMT and subsequent chemotherapeutic resistance.

There are a variety of EMT-driven mechanisms that lead to the acquisition of chemoresistance such as lower drug uptake, higher drug efflux, higher DNA repair capacity, and decreased apoptosis (Loret et al., 2019). In our study, the results of mRNA microarray analysis showed that platinum drug-resistance genes including ERBB2, BCL-2, MAP3K5, PIK3R1, and BIRC3 are significantly upregulated in SKOV-3 cells on soft substrates. Avian erythroblastosis oncogene B2 (ERBB2) also known as human epidermal growth factor receptor 1 (HER2) signaling is highly correlated with cisplatin-resistance in OCCs and tumors (Harris et al., 2019). BCL-2, anti-apototic protein, can block p53-mediated apoptosis (Kassim et al., 1999; Dai et al., 2017) and involve in AKT-regulated cell survival in cisplatin resistant EOC (Dai et al., 2017). It is a potential predictor of cisplatin-resistance in EOC. ABC transporters exclude drugs from the cytoplasm and move them to the extracellular environment using the energy provided by ATP hydrolysis. EMT transcription factors can induce the expression of ABC transporters. ABCB1 (MDR1), encoding p-glycoprotein (PgP) is the most studied ABC transporter and the first to be identified to selectively confer MDR by directly pumping out anticancer drugs, including paclitaxel, doxorubicin, topotecan, docetaxel (Juliano and Ling, 1976; Bourhis et al., 1989; Veneroni et al., 1994). Recent studies indicate that expression of ABCB1 is a useful predictor of paclitaxel resistant for patients with ovarian cancer (Kamazawa et al., 2002; Haque et al., 2020). ABCB1 was expressed at higher levels in more mesenchymal and therapy-resistant OCC lines, than in more epithelial and chemo-sensitive cell lines (Feng et al., 2017; Zhang et al., 2018). Furthermore, ABCB4 (MDR3) is also linked with chemotherapy resistance and is increased in recurrent ovarian cancers (Duan et al., 2004). Paclitaxel-resistant cell lines overexpress both ABCB1 and ABCB4 (Januchowski et al., 2014). Correlation analyses showed a high correlation between MDR3 expression and resistance to paclitaxel and doxorubicin *in vitro*. Our data showed that cells cultured on softer gels expressed more ABCB1 and ABCB4, which coincidentally were more mesenchymal and therapy-resistant. These results suggest that EMT promotes chemoresistance in SKOV-3 cells on soft substrates via the upregulation of ABCB1 and ABCB4.

## Conclusion

In conclusion, our study showed that increasing substrate stiffness promotes the proliferation of SKOV-3 cells and the nuclear localization of YAP. Conversely, a soft environment (as might be encountered by disseminated or metastatic OCCs) induces EMT and chemoresistance. Chemoresistance in SKOV-3 cells on softer substrates was due to the upregulation of platinum drug-resistant genes, ABCB1, and ABCB4. These findings provide new therapeutic targets for future anti-cancer drug designs.

## Data Availability Statement

The data presented in this study are deposited in https://www.ncbi.nlm.nih.gov/geo/query/acc.cgi?acc=GSE178888.

## Author Contributions

YF: cell culture, western immunoblotting, data analysis, and manuscript writing. QS: research supervision, cell culture, proliferation and cytotoxicity analysis, AFM experimental implementation, statistical analysis of data, manuscript writing, and critical review and revision. XLa: cell culture, immunofluorescence staining, cell staining, and ESEM imaging. JF: technical adviser, data analysis, and statistical analysis of data. ZA: MATLAB code writing and data analysis. XLn: mRNA microarray analysis. JW: research supervision, and manuscript critical review and revision. All the authors read and approved the final manuscript.

## Conflict of Interest

The authors declare that the research was conducted in the absence of any commercial or financial relationships that could be construed as a potential conflict of interest. The handling editor declared a past co-authorship with one of the authors JF.

## Publisher’s Note

All claims expressed in this article are solely those of the authors and do not necessarily represent those of their affiliated organizations, or those of the publisher, the editors and the reviewers. Any product that may be evaluated in this article, or claim that may be made by its manufacturer, is not guaranteed or endorsed by the publisher.

## References

[B1] Alibert C. Goud B. Manneville J. (2017). Are cancer cells really softer than normal cells? *Biol. Cell* 109 167–189. 10.1111/boc.201600078 28244605

[B2] Aragona M. Panciera T. Manfrin A. Giulitti S. Michielin F. Elvassore N. (2013). A mechanical checkpoint controls multicellular growth through YAP/TAZ regulation by actin-processing factors. *Cell* 154 1047–1059. 10.1016/j.cell.2013.07.042 23954413

[B3] Beil M. Micoulet A. von Wichert G. Paschke S. Walther P. Omary M. B. (2003). Sphingosylphosphorylcholine regulates keratin network architecture and visco-elastic properties of human cancer cells. *Nat. Cell Biol.* 5 803–811. 10.1038/ncb1037 12942086

[B4] Benham-Pyle B. W. Pruitt B. L. Nelson W. J. (2015). Mechanical strain induces E-cadherin-dependent Yap1 and β-catenin activation to drive cell cycle entry. *Science* 348 1024–1027. 10.1126/science.aaaa4559 26023140 PMC4572847

[B5] Bourhis J. Goldstein L. J. Riou G. Pastan I. Gottesman M. M. Bénard J. (1989). Expression of a human multidrug resistance gene in ovarian carcinomas. *Cancer Res.* 49 5062–5065. 2766278

[B6] Correia A. L. Bissell M. J. (2012). The tumor microenvironment is a dominant force in multidrug resistance. *Drug Resist. Updat.* 15 39–49. 10.1016/j.drup.2012.01.006 22335920 PMC3658318

[B7] Dai Y. Jin S. Li X. Wang D. (2017). The involvement of Bcl-2 family proteins in AKT-regulated cell survival in cisplatin resistant epithelial ovarian cancer. *Oncotarget* 8 1354–1368. 10.18632/oncotarget.13817 27935869 PMC5352061

[B8] De Craene B. Berx G. (2013). Regulatory networks defining EMT during cancer initiation and progression. *Nat. Rev. Cancer* 13 97–110. 10.1038/nrc3447 23344542

[B9] Duan Z. Brakora K. A. Seiden M. V. (2004). Inhibition of ABCB1 (MDR1) and ABCB4 (MDR3) expression by small interfering RNA and reversal of paclitaxel resistance in human ovarian cancer cells. *Mol. Cancer Ther.* 3 833–838. 10.1016/j.lungcan.2004.01.008 15252144

[B10] Dupont S. Morsut L. Aragona M. Enzo E. Giulitti S. Cordenonsi M. (2011). Role of YAP/TAZ in mechanotransduction. *Nature* 474 179–183. 10.1038/nature10137 21654799

[B11] Erkan M. Hausmann S. Michalski C. W. Fingerle A. A. Dobritz M. Kleeff J. (2012). The role of stroma in pancreatic cancer: diagnostic and therapeutic implications. *Nat. Rev. Gastroenterol. Hepatol.* 9 454–467. 10.1038/nrgastro.2012.115 22710569

[B12] Feng J. Gou J. Jia J. Yi T. Cui T. Li Z. (2016). Verteporfin, a suppressor of YAP-TEAD complex, presents promising antitumor properties on ovarian cancer. *Onco. Targets Ther.* 9 5371–5381. 10.2147/ott.S109979 27621651 PMC5010158

[B13] Feng J. T. Tang Y. Xu Y. G. Sun Q. M. Liao F. L. Han D. (2013). Substrate stiffness influences the outcome of antitumor drug screening *in vitro*. *Clin. Hemorheol. Micro.* 55 121–131. 10.3233/Ch-131696 23445634

[B14] Feng T. Wang Y. Lang Y. Zhang Y. (2017). KDM5A promotes proliferation and EMT in ovarian cancer and closely correlates with PTX resistance. *Mol. Med. Rep.* 16 3573–3580. 10.3892/mmr.2017.6960 28714030

[B15] Fletcher D. A. Mullins R. D. (2010). Cell mechanics and the cytoskeleton. *Nature* 463 485–492. 10.1038/nature08908 20110992 PMC2851742

[B16] Florea A. M. Büsselberg D. (2011). Cisplatin as an anti-tumor drug: cellular mechanisms of activity, drug resistance and induced side effects. *Cancers* 3 1351–1371. 10.3390/cancers3011351 24212665 PMC3756417

[B17] Fraser M. Bai T. Tsang B. K. (2008). Akt promotes cisplatin resistance in human ovarian cancer cells through inhibition of p53 phosphorylation and nuclear function. *Int. J. Cancer* 122 534–546. 10.1002/ijc.23086 17918180

[B18] Galluzzi L. Senovilla L. Vitale I. Michels J. Martins I. Kepp O. (2012). Molecular mechanisms of cisplatin resistance. *Oncogene* 31 1869–1883. 10.1038/onc.2011.384 21892204

[B19] Haque A. Sait K. Alam Q. Alam M. Z. Rasool M. (2020). MDR1 Gene polymorphisms and its association with expression as a clinical relevance in terms of response to chemotherapy and prognosis in ovarian cancer. *Front. Genet.* 11:516. 10.3389/fgene.2020.00516 32528530 PMC7264409

[B20] Harris F. R. Zhang P. Yang L. Hou X. Leventakos K. Weroha S. J. (2019). Targeting HER2 in patient-derived xenograft ovarian cancer models sensitizes tumors to chemotherapy. *Mol. Oncol.* 13 132–152. 10.1002/1878-0261.12414 30499260 PMC6360362

[B21] Hazlehurst L. A. Argilagos R. F. Dalton W. S. (2007). Beta1 integrin mediated adhesion increases Bim protein degradation and contributes to drug resistance in leukaemia cells. *Br. J. Haematol.* 136 269–275. 10.1111/j.1365-2141.2006.06435.x 17233818

[B22] Hazlehurst L. A. Damiano J. S. Buyuksal I. Pledger W. J. Dalton W. S. (2000). Adhesion to fibronectin via beta1 integrins regulates p27kip1 levels and contributes to cell adhesion mediated drug resistance (CAM-DR). *Oncogene* 19 4319–4327. 10.1038/sj.onc.1203782 10980607

[B23] Horwitz S. B. (1992). Mechanism of action of taxol. *Trends Pharmacol. Sci.* 13 134–136. 10.1016/0165-6147(92)90048-b 1350385

[B24] Huang J. Wu S. Barrera J. Matthews K. Pan D. (2005). The Hippo signaling pathway coordinately regulates cell proliferation and apoptosis by inactivating Yorkie, the Drosophila Homolog of YAP. *Cell* 122 421–434. 10.1016/j.cell.2005.06.007 16096061

[B25] Hui L. Zhang J. Ding X. Guo X. Jiang X. (2017). Matrix stiffness regulates the proliferation, stemness and chemoresistance of laryngeal squamous cancer cells. *Int. J. Oncol.* 50 1439–1447. 10.3892/ijo.2017.3877 28259905

[B26] Januchowski R. Wojtowicz K. Andrzejewska M. Zabel M. (2014). Expression of MDR1 and MDR3 gene products in paclitaxel-, doxorubicin- and vincristine-resistant cell lines. *Biomed. Pharmacother.* 68 111–117. 10.1016/j.biopha.2013.09.004 24140176

[B27] Jemal A. Siegel R. Xu J. Ward E. (2010). Cancer statistics, 2010. *CA Cancer J Clin* 60 277–300. 10.3322/caac.20073 20610543

[B28] Juliano R. L. Ling V. (1976). A surface glycoprotein modulating drug permeability in chinese hamster ovary cell mutants. *Biochim. Biophys. Acta*. 455 152–162. 10.1016/0005-2736(76)90160-7 990323

[B29] Kajiyama H. Shibata K. Terauchi M. Yamashita M. Ino K. Nawa A. (2007). Chemoresistance to paclitaxel induces epithelial-mesenchymal transition and enhances metastatic potential for epithelial ovarian carcinoma cells. *Int. J. Oncol.* 31 277–283. 10.3892/ijo.31.2.277 17611683

[B30] Kamazawa S. Kigawa J. Kanamori Y. Itamochi H. Sato S. Iba T. (2002). Multidrug resistance gene-1 is a useful predictor of paclitaxel-based chemotherapy for patients with ovarian cancer. *Gynecol. Oncol.* 86 171–176. 10.1006/gyno.2002.6738 12144824

[B31] Kapoor A. Barai A. Thakur B. Das A. Patwardhan S. R. Monteiro M. (2018). Soft drugresistant ovarian cancer cells migrate via two distinct mechanisms utilizing myosin II based contractility. *Biochim. Biophys. Acta.* 1865 392–405. 10.1016/j.bbamcr.2017.11.012 29175377

[B32] Kassim S. K. Ali H. S. Sallam M. M. Fayed S. T. Seada L. S. Abd-Elkawy E. (1999). Increased bcl-2 expression is associated with primary resistance to chemotherapy in human epithelial ovarian cancer. *Clin. Biochem.* 32 333–338. 10.1016/s0009-9120(99)00026-0 10480447

[B33] Kharaishvili G. Simkova D. Bouchalova K. Gachechiladze M. Narsia N. Bouchal J. (2014). The role of cancer-associated fibroblasts, solid stress and other microenvironmental factors in tumor progression and therapy resistance. *Cancer Cell Int.* 14:41. 10.1186/1475-2867-14-41 24883045 PMC4038849

[B34] Landowski T. H. Olashaw N. E. Agrawal D. Dalton W. S. (2003). Cell adhesion-mediated drug resistance (CAM-DR) is associated with activation of NF-kappa B (RelB/p50) in myeloma cells. *Oncogene* 22 2417–2421. 10.1038/sj.onc.1206315 12717418

[B35] Lekka M. (2016). Discrimination between normal and cancerous cells using AFM. *Bionanoscience* 6 65–80. 10.1007/s12668-016-0191-3 27014560 PMC4778153

[B36] Levental I. Georges P. C. Janmey P. A. (2007). Soft biological materials and their impact on cell function. *Soft Matter* 3 299–306. 10.1039/b610522j 32900146

[B37] Levental K. R. Yu H. Kass L. Lakins J. N. Egeblad M. Erler J. T. (2009). Matrix crosslinking forces tumor progression by enhancing integrin signaling. *Cell* 139 891–906. 10.1016/j.cell.2009.10.027 19931152 PMC2788004

[B38] Li L. Xu Q. H. Dong Y. H. Li G. X. Li H. Y. (2016). MiR-181a upregulation is associated with epithelial-To-mesenchymal transition (EMT) and multidrug resistance (MDR) of ovarian cancer cells. *Eur. Rev. Med. Pharmacol. Sci.* 20 2004–2010. 27249598

[B39] Li Q. S. Lee G. Ong C. N. Lim C. T. (2008). AFM indentation study of breast cancer cells. *Biochem. Biophys. Res. Commun.* 374 609–613. 10.1016/j.bbrc.2008.07.078 18656442

[B40] Liu C. Liu Y. Xie H. G. Zhao S. Xu X. X. Fan L. X. (2015). Role of three-dimensional matrix stiffness in regulating the chemoresistance of hepatocellular carcinoma cells. *Biotechnol. Appl. Biochem.* 62 556–562. 10.1002/bab.1302 25274163

[B41] Liu C. Y. Li X. Hua W. D. Li J. J. Han X. X. Ha Q. (2016). Porous matrix stiffness modulates response to targeted therapy in breast carcinoma. *Small* 12 4675–4681. 10.1002/smll.201601365 27295361

[B42] Liu-Chittenden Y. Huang B. Shim J. S. Chen Q. Lee S. J. Anders R. A. (2012). Genetic and pharmacological disruption of the TEAD-YAP complex suppresses the oncogenic activity of YAP. *Genes Dev.* 26 1300–1305. 10.1101/gad.192856.112 22677547 PMC3387657

[B43] Loret N. Denys H. Tummers P. Berx G. (2019). The role of epithelial-to-mesenchymal plasticity in ovarian cancer progression and therapy resistance. *Cancers* 11:838. 10.3390/cancers11060838 31213009 PMC6628067

[B44] McKenzie A. J. Hicks S. R. Svec K. V. Naughton H. Edmunds Z. L. Howe A. K. (2018). The mechanical microenvironment regulates ovarian cancer cell morphology, migration, and spheroid disaggregation. *Sci. Rep.* 8:7228. 10.1038/s41598-018-25589-0 29740072 PMC5940803

[B45] Mor G. Alvero A. (2013). The duplicitous origin of ovarian cancer. *Rambam Maimonides Med. J.* 4:e0006. 10.5041/RMMJ.10106 23908856 PMC3678912

[B46] Nieman K. M. Kenny H. A. Penicka C. V. Ladanyi A. Buell-Gutbrod R. Zillhardt M. R. (2011). Adipocytes promote ovarian cancer metastasis and provide energy for rapid tumor growth. *Nat. Med.* 17 1498–1503. 10.1038/nm.2492 22037646 PMC4157349

[B47] Ostman A. (2012). The tumor microenvironment controls drug sensitivity. *Nat. Med.* 18 1332–1334. 10.1038/nm.2938 22961158

[B48] Park S. (2016). Nano-mechanical phenotype as a promising biomarker to evaluate cancer development, progression, and anti-cancer drug efficacy. *J. Cancer Prev.* 21 73–80. 10.15430/JCP.2016.21.2.73 27390735 PMC4933430

[B49] Piccolo S. Dupont S. Cordenonsi M. (2014). The biology of YAP/TAZ: hippo signaling and beyond. *Physiol. Rev.* 94 1287–1312. 10.1152/physrev.00005.2014 25287865

[B50] Ren J. Huang H. Liu Y. Zheng X. Zou Q. (2015). An atomic force microscope study revealed two mechanisms in the effect of anticancer drugs on rate-dependent young’s modulus of human prostate cancer cells. *PLoS One* 10:e0126107. 10.1371/journal.pone.0126107 25932632 PMC4416805

[B51] Rice A. J. Cortes E. Lachowski D. Cheung B. C. H. Karim S. A. Morton J. P. (2017). Matrix stiffness induces epithelial–mesenchymal transition and promotes chemoresistance in pancreatic cancer cells. *Oncogenesis* 6:e352. 10.1038/oncsis.2017.54 28671675 PMC5541706

[B52] Schrader J. Gordon-Walker T. T. Aucott R. L. van Deemter M. Quaas A. Walsh S. (2011). Matrix stiffness modulates proliferation, chemotherapeutic response, and dormancy in hepatocellular carcinoma cells. *Hepatology* 53 1192–1205. 10.1002/hep.24108 21442631 PMC3076070

[B53] Seo Y. H. Jo Y. N. Oh Y. J. Park S. (2015). Nano-mechanical reinforcement in drug-resistant ovarian cancer cells. *Biol. Pharm. Bull.* 38 389–395. 10.1248/bpb.b14-00604 25757920

[B54] Sethi T. Rintoul R. C. Moore S. M. MacKinnon A. C. Salter D. Choo C. (1999). Extracellular matrix proteins protect small cell lung cancer cells against apoptosis: a mechanism for small cell lung cancer growth and drug resistance *in vivo*. *Nat. Med.* 5 662–668. 10.1038/9511 10371505

[B55] Sharma S. Santiskulvong C. Rao J. Gimzewski J. K. Dorigo O. (2014). The role of Rho GTPase in cell stiffness and cisplatin resistance in ovarian cancer cells. *Integr. Biol.* 6 611–617. 10.1039/c3ib40246k 24718685

[B56] Shi L. Shi S. Li J. Sun Q. Feng K. Chen P. (2010). AFM and fluorescence imaging of nanomechanical response in periodontal ligament cells. *Front. Biosci.* 2:1028–41. 10.2741/e161 20515773

[B57] Tajik A. Zhang Y. J. Wei F. X. Sun J. Jia Q. Zhou W. W. (2016). Transcription upregulation via force-induced direct stretching of chromatin. *Nat. Mater.* 15 1287–1296. 10.1038/Nmat4729 27548707 PMC5121013

[B58] Taube J. H. Herschkowitz J. I. Komurov K. Zhou A. Y. Gupta S. Yang J. (2010). Core epithelial-to-mesenchymal transition interactome gene-expression signature is associated with claudin-low and metaplastic breast cancer subtypes. *Proc. Natl. Acad. Sci. U. S. A.* 107 15449–15454. 10.1073/pnas.1004900107 20713713 PMC2932589

[B59] Thompson E. W. Newgreen D. F. Tarin D. (2005). Carcinoma invasion and metastasis: a role for epithelial-mesenchymal transition? *Cancer Res.* 65 5991–5995. 10.1158/0008-5472.CAN-05-0616 16024595

[B60] Tilghman R. W. Cowan C. R. Mih J. D. Koryakina Y. Gioeli D. Slack-Davis J. K. (2010). Matrix rigidity regulates cancer cell growth and cellular phenotype. *PLoS One* 5:e12905. 10.1371/journal.pone.0012905 20886123 PMC2944843

[B61] Totaro A. Castellan M. Battilana G. Zanconato F. Azzolin L. Giulitti S. (2017). YAP/TAZ link cell mechanics to notch signalling to control epidermal stem cell fate. *Nat. Commun.* 8:15206. 10.1038/ncomms15206 28513598 PMC5442321

[B62] Veneroni S. Zaffaroni N. Daidone M. G. Benini E. Villa R. Silvestrini R. (1994). Expression of p-glycoprotein and *in vitro* or *in vivo* resistance to doxorubicin and cisplatin in breast and ovarian cancers. *Eur. J. Cancer* 30A 1002–1007. 10.1016/0959-8049(94)90132-5 7946563

[B63] Walker J. L. Powell C. B. Chen L. M. Carter J. Bae Jump V. L. Parker L. P. (2015). Society of gynecologic oncology recommendations for the prevention of ovarian cancer. *Cancer* 121 2108–2120. 10.1002/cncr.29321 25820366

[B64] Watanabe O. Imamura H. Shimizu T. Kinoshita J. Okabe T. Hirano A. (2004). Expression of twist and wnt in human breast cancer. *Anticancer Res.* 24 3851–3856. 15736421

[B65] Wu H. W. Kuhn T. Moy V. T. (1998). Mechanical properties of L929 cells measured by atomic force microscopy: effects of anticytoskeletal drugs and membrane crosslinking. *Scanning* 20 389–397. 10.1002/sca.1998.4950200504 9737018

[B66] Wu P. Gao W. Su M. Nice E. C. Zhang W. Lin J. (2021). Adaptive mechanisms of tumor therapy resistance driven by tumor microenvironment. *Front Cell Dev. Biol.* 9:641469. 10.3389/fcell.2021.641469 33732706 PMC7957022

[B67] Xu W. Roman M. Byungkyu K. Wang L. John M. Todd S. (2012). Cell stiffness is a biomarker of the metastatic potential of ovarian cancer cells. *PLoS One* 7:e46609. 10.1371/journal.pone.0046609 23056368 PMC3464294

[B68] Yang J. Mani S. A. Donaher J. L. Ramaswamy S. Itzykson R. A. Come C. (2004). Twist, a master regulator of morphogenesis, plays an essential role in tumor metastasis. *Cell* 117 927–939. 10.1016/j.cell.2004.06.006 15210113

[B69] Yang J. Zaman M. M. Vlasakov I. Roy R. Huang L. Martin C. R. (2019). Adipocytes promote ovarian cancer chemoresistance. *Sci. Rep.* 9:13316. 10.1038/s41598-019-49649-1 31527632 PMC6746782

[B70] Yang Q. Huang J. Wu Q. Cai Y. Zhu L. Lu X. (2014). Acquisition of epithelial-mesenchymal transition is associated with Skp2 expression in paclitaxel-resistant breast cancer cells. *Br. J. Cancer* 110 1958–1967. 10.1038/bjc.2014.136 24642627 PMC3992499

[B71] Yang X. Fraser M. Moll U. M. Basak A. Tsang B. K. (2006). Akt-mediated cisplatin resistance in ovarian cancer: modulation of p53 action on caspase-dependent mitochondrial death pathway. *Cancer Res.* 66 3126–3136. 10.1158/0008-5472.Can-05-0425 16540663

[B72] Zhang Y. Huang S. Guo Y. Li L. (2018). MiR-1294 confers cisplatin resistance in ovarian cancer cells by targeting IGF1R. *Biomed. Pharmacother.* 106 1357–1363. 10.1016/j.biopha.2018.07.059 30119207

[B73] Zhao B. Li L. Wang L. Wang C. Y. Yu J. Guan K. L. (2012). Cell detachment activates the Hippo pathway via cytoskeleton reorganization to induce anoikis. *Genes Dev.* 26 54–68. 10.1101/gad.173435.111 22215811 PMC3258966

[B74] Zhu X. Shen H. Yin X. Long L. Xie C. Liu Y. (2016). MiR-186 regulation of Twist1 and ovarian cancer sensitivity to cisplatin. *Oncogene* 35 323–332. 10.1038/onc.2015.84 25867064

